# A brief exposure to toluene vapor alters the intrinsic excitability of D2 medium spiny neurons in the rat ventral striatum

**DOI:** 10.3389/fnins.2023.1235866

**Published:** 2023-08-03

**Authors:** Michael Okas, Abigail Kastner, Dominic Gioia, John J. Woodward

**Affiliations:** Department of Neuroscience, Medical University of South Carolina, Charleston, SC, United States

**Keywords:** inhalants, nucleus accumbens, action potentials, slice electrophysiology, medium spiny neurons (MSN)

## Abstract

Although volatile organic solvents such as toluene are used for commercial and industrial uses, they are often voluntarily inhaled for their intoxicating and euphoric effects. Research into the effects of inhalants such as toluene on brain function have revealed actions on a variety of ligand-gated and voltage-activated ion channels involved in regulating neuronal excitability. Previous work from this laboratory has also shown that brief exposures to toluene vapor induce changes in the intrinsic excitability and synaptic transmission of neurons within the medial prefrontal cortex and ventral tegmental area that vary depending on projection target. In the present study, we recorded current-evoked spiking of medium spiny neurons (MSNs) in the nucleus accumbens (NAc) core and shell in adolescent rats exposed to an intoxicating concentration of toluene vapor. Compared to air controls, firing of NAc core MSNs in Sprague–Dawley rats was not altered 24 h after exposure to 10,500 ppm toluene vapor while spiking of NAc shell MSNs was enhanced at low current steps but reduced at higher current steps. When the rheobase current was used to putatively identify MSN subtypes, both “D1-like” and “D2-like” MSNs within the NAc shell but not core showed toluene-induced changes in firing. As toluene may itself have altered the rheobase resulting in misclassification of neuron subtype, we conducted additional studies using adolescent D2-Cre rats infused with a Cre-dependent mCherry reporter virus. Following toluene vapor exposure, spiking of NAc shell D2+ MSNs was enhanced at low current steps but inhibited at higher currents as compared to air controls while there were no differences in the firing of NAc shell D2- MSNs. The toluene-induced change in NAc D2+ shell MSN firing was accompanied by alterations in membrane resistance, rheobase, action potential rise time and height with no changes noted in D2- MSNs. Overall, these data add to a growing literature showing that brief exposures to intoxicating concentrations of toluene vapor causes selective alterations in the excitability of neurons within the addiction neurocircuitry that vary depending on sub-region, cell-type and projection target.

## Introduction

Volatile organic solvents such as toluene (methylbenzene) are found in a wide variety of industrial and household products such as paint thinners, adhesives and degreasers. In addition to these commercial uses, volatile organic solvents are often voluntarily inhaled to produce intoxication and feelings of euphoria (Balster, 2019). Inhalant use is relatively common with over 26 million Americans reporting having used inhalants at least once over their lifetime with the highest prevalence among children and adolescents 12–17 years old (SAMHSA, 2021). Inhalant use is also common worldwide with higher rates of use reported for street children (Praveen et al., 2012), native populations (Kaufman, 1973; Beauvais et al., 2002; Cairney et al., 2002; Vazan et al., 2011) and those living in rural, poor or isolated areas (Kann et al., 2016). While the use of inhalants usually declines with age (SAMHSA, 2021), exposure during childhood or adolescence increases the risk of developing a substance use disorder later in life (Wu and Ringwalt, 2006).

The mechanisms that drive the intoxicating and rewarding effects of toluene are not completely understood but likely reflect alterations in brain activity in areas involved in goal-directed behavior (reviewed by Cruz and Bowen, 2021; Woodward and Braunscheidel, 2023). For example, a brief exposure of adolescent rats to toluene vapor induced an increase in the AMPA/NMDA ratio of dopamine neurons in the ventral tegmental area (VTA) that project to the nucleus accumbens (NAc) but no change in those projecting to the medial prefrontal cortex (mPFC) similar to that reported for other abused drugs (Lammel et al., 2011; Beckley et al., 2013). Repeated exposures to toluene vapor induces a conditioned place preference (CPP) in rodents (Funada et al., 2002; Wayman and Woodward, 2018a) while rats, mice and monkeys can learn to self-administer toluene vapor (Wood et al., 1977; Blokhina et al., 2004; Braunscheidel et al., 2020). In the CPP study from our laboratory, toluene exposure produced selective effects on the excitability of mPFC neurons projecting to different areas of the NAc and the expression of conditioned place preference was blocked by chemogenetic excitation of mPFC neurons that project to the NAc shell (Wayman and Woodward, 2018a). An important unanswered question is whether exposure to toluene vapor also alters the excitability of NAc neurons and whether this shows a similar degree of selectivity.

Over 95% of the neurons in the NAc are GABAergic medium spiny neurons (MSN) and these are equally divided between those that primarily express the D1 or the D2 dopamine receptor (Matamales et al., 2009). Both D1 and D2 MSNs are located in the core and shell of the nucleus accumbens and are thought to be critically involved in the control of motivated behavior including seeking and taking of rewarding substances (Allichon et al., 2021). In the present study, we exposed adolescent rats to an intoxicating concentration of toluene vapor that mimics human solvent inhalation and measured the intrinsic excitability of NAc MSNs. As discussed below, toluene induced changes in the excitability of NAc shell but not core neurons and this was restricted to D2 MSNs.

## Materials and methods

### Animals

Two different strains of rats were used in the experiments described below. In the first study, we used male Sprague–Dawley rats purchased from Envigo RMS (Indianapolis, IN) that arrived at MUSC at post-natal day (PND) 21. The second study used male transgenic rats derived from a breeding colony established with hemizygous male and female D2-Cre rats generated on a Long-Evans background (RRRC #00768; Columbia, MO). Offspring were genotyped using tail snips obtained at PND 21 with primers for the Drd2 promoter (forward primer; 5’-TCA GGG AAC CCT CTT TGA GA-3′) and Cre recombinase (reverse primer; 5’-CAC AGT CAG CAG GTT GGA GA-3′). In both studies, rats were housed in pairs with free access to food and water in a temperature and humidity-controlled animal facility operated under a reverse 12 h light/dark cycle (Off 9:00 AM; On 9:00 PM). All studies were performed in accordance with protocols approved by the MUSC Institutional Animal Care and Use Committee.

### Rat viral surgery

D2-Cre rats underwent stereotactic surgery at PND 28–30 to label D2 MSNs. Rats were initially anesthetized with 3% isoflurane and this was reduced to 1–2% isoflurane throughout the surgery. Body temperature was maintained with a heating pad. After mounting in a stereotaxic rig, rats were given an injection of carprofen (2.5 mg/kg, i.p.) and the Cre-dependent virus AAV1-hSyn-DIO-mCherry (Addgene #50459, Watertown, MA) was bilaterally infused (300 nL per side, 60 nL/min) into the ventral striatum (in mm from bregma: A/P + 2.0; M/L 0.8–1.4; D/V -6.0). Following the infusion, the injector was left in place for an additional 5 min to allow for diffusion of the virus. After surgery, the rat was returned to the housing facility and monitored until further use.

### Toluene vapor exposure

Adolescent (PND 40–43) rats were exposed to air or toluene vapor in a dark gray anesthesia chamber (30x30x30 cm; Plas Labs, Lansing, MI) containing an inlet and outlet and a raised floor mounted approximately 2 cm above the bottom of the chamber. The chamber was located inside a chemical fume hood equipped with a light blocking curtain and an air flow of 100 *cf.* On day 1, rats were placed in the chamber for 1 hour with air supplied at a flow rate of 4 L/min and then returned to the housing facility. On day 2, rats received either a 1 h exposure to air (controls) or toluene (methlybenzene, Sigma-Aldrich, St Louis, MO) and then were returned to the housing facility. For the toluene exposure, air was delivered to the chamber for the first 10 min followed by two 10 min exposures to toluene vapor (10,500 ppm each) separated by a 10 min air purge and a final 20 min air washout. Toluene vapor was delivered to the chamber via a sevoflurane vaporizer and vapor concentrations within the chamber were verified using a portable toluene vapor detector (DOD Technologies, Cary, IL) as previously described (Wayman and Woodward, 2018b).

### Slice electrophysiology

Twenty-four hours after the air or toluene vapor exposure, rats were anesthetized with urethane (3 mg/kg, i.p.). Coronal sections (260 um) of the brain containing the ventral striatum were prepared with a Leica VT1200S vibratome (Leica Biosystems, Buffalo Grove, IL) using ice-cold, oxygenated (95% O_2_, 5% CO_2_) sucrose-substituted cutting solution containing (in mm): 200 sucrose, 1.9 KCl, 6 MgSO_4_, 1.4 NaH_2_PO_4_, 25 NaHCO_3_, 0.5 CaCl_2_, 10 glucose, and 0.4 ascorbic acid; pH 7.35–7.45 with 310–320 mOsm. Slices were then transferred to a holding chamber filled with oxygenated artificial cerebrospinal fluid (aCSF) containing (in mM): 125 NaCl, 2.5 KCl, 25 NaHCO_3_, 1.4 NaH_2_PO_4_, 1.3 MgCl_2_, 2 CaCl_2_, and 10 glucose; pH 7.35–7.45, with 310–320 mOsm (Beckley et al., 2013). Brain slices were incubated at 34°C for 30 min and then held at room temperature until use.

For recordings, slices were transferred to a recording chamber and perfused with oxygenated and heated (~34°C) aCSF at a flow rate of 3 mL/min using inline and bath heaters (Warner Instruments, Holliston, MA). Neurons within the ventral striatum were visualized using a Zeiss FS2 microscope (Zeiss, Thorndale, NY) equipped with fluorescent optics, infrared Dodt-gradient contrast illumination (Luigs and Neuman, Ratingen, Germany) and an infrared camera (IR-1000; Dage-MTI, Michigan City, IN). Pipettes were prepared from thin-wall borosilicate glass (OD = 1.5 mm, ID = 1.1 mm) on a Sutter P97 micropipette puller (Novato, CA) with tip resistances between 2.5–5 megaohms. They were filled with recording solution consisting of (in mM): 120 K-gluconate, 10 HEPES, 10 KCl, 2 MgCl_2_, 2 Na_2_ATP, 0.3 NaGTP, 1 EGTA and 0.2% biocytin; pH 7.35–7.45, and 285–295 mOsm. A gigaohm seal was obtained under voltage-clamp mode followed by suction to achieve breakthrough and whole-cell access. Neurons were held at −80 mV and test pulses were given to determine membrane and series resistance. Neurons were not used if series resistance was initially greater than 20 mOhm or if it changed by more than 25% during the recording. The amplifier was switched to I = 0 mode to record resting membrane potential and then to current-clamp mode with current adjusted to bring the membrane potential to −80 mV. Resting membrane potentials were not corrected for the liquid junction potential error (~12.2 mV). Spike firing was induced by a series of current injections (0–440 pA; 750 msec each) delivered through the patch pipette. All recordings were performed using an Axon MultiClamp 700A amplifier (Molecular Devices, Union City, CA) and Instrutech ITC-18 analog-digital converter (HEKA Instruments, Bellmore, NY) controlled by AxographX software (Axograph, Sydney, Australia) running on a Macintosh G4 computer (Apple, Cupertino, CA). Data were filtered at 4 kHz and acquired at a sampling rate of 10 kHz.

### Data analysis

Recordings were analyzed for spike number and action potential (AP) parameters using AxographX software and included action potential height, rise-time, half-width, spike threshold and after-hyperpolarization (AHP). AHP was measured as the difference between the spike threshold and the most negative potential during the hyperpolarization. All data are presented as mean ± SEM and were analyzed by Prism software (GraphPad, San Diego, CA) using two-way Anova and unpaired *t*-tests where indicated. Values were considered significantly different when *p* < 0.05.

## Results

**Figure 1:**
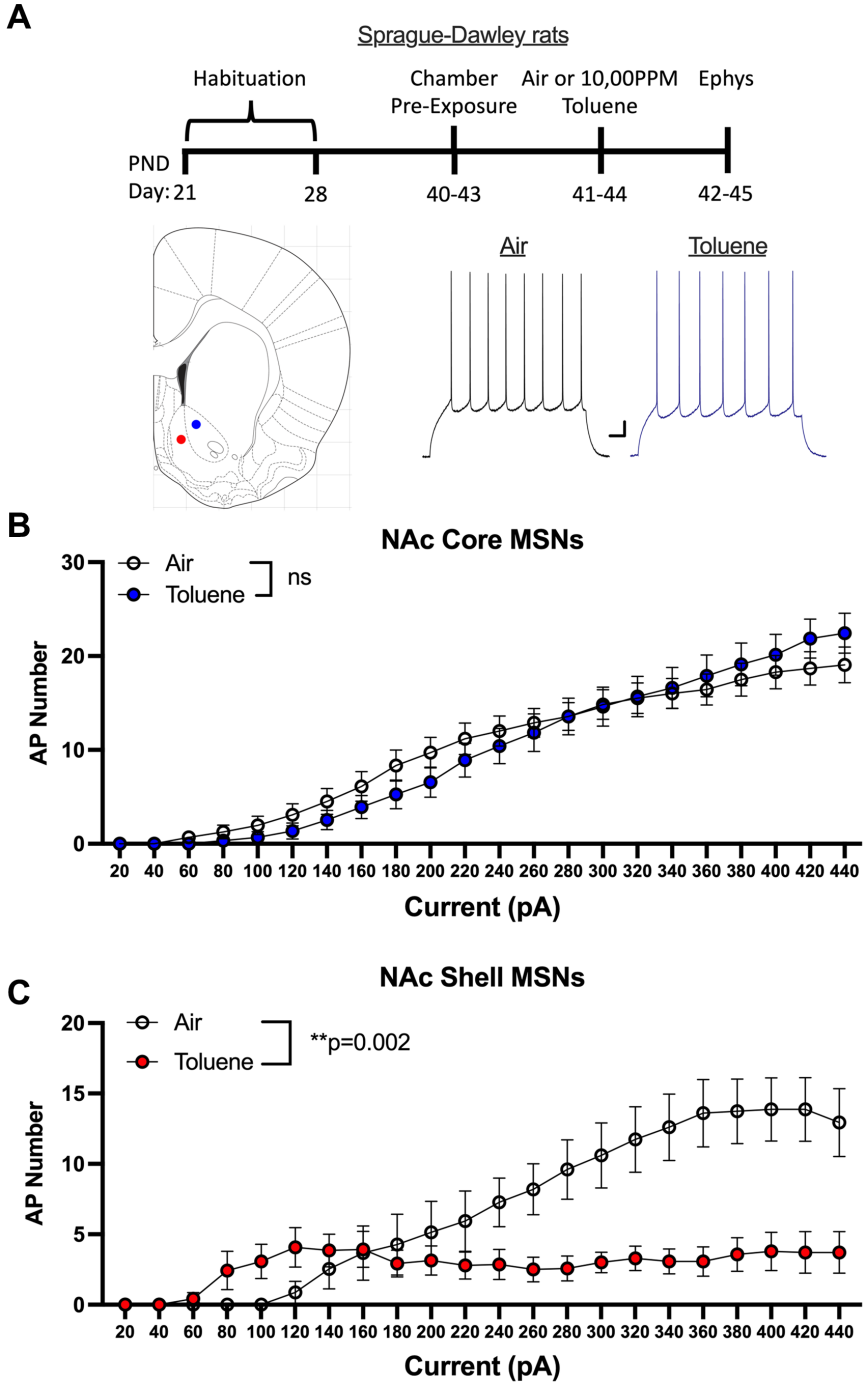
Exposure to toluene vapor alters the excitability of NAc neurons in adolescent Sprague–Dawley rats. **(A)** Schematic shows timeline of study and age of rats at toluene exposure and electrophysiology recordings. Inset shows approximate location of recordings for NAc core (blue dot) and shell (red dot) MSNs. Traces show examples of current evoked spiking of NAc core MSNs from air or toluene exposed rats. Scale bars: X-axis 0.1 s; Y-axis 10 mV. **(B)** Current evoked spiking of NAc core MSNs was not different between air and toluene exposed rats. **(C)** Toluene exposure enhanced current-evoked spiking of NAc shell MSNs at low current steps and reduced spiking at higher current amplitudes as compared to air controls (RM two-way Anova *F*(_1,27_) = 11.65, *p* = 0.002). Data are presented as mean ± sem.

**Figure 2:**
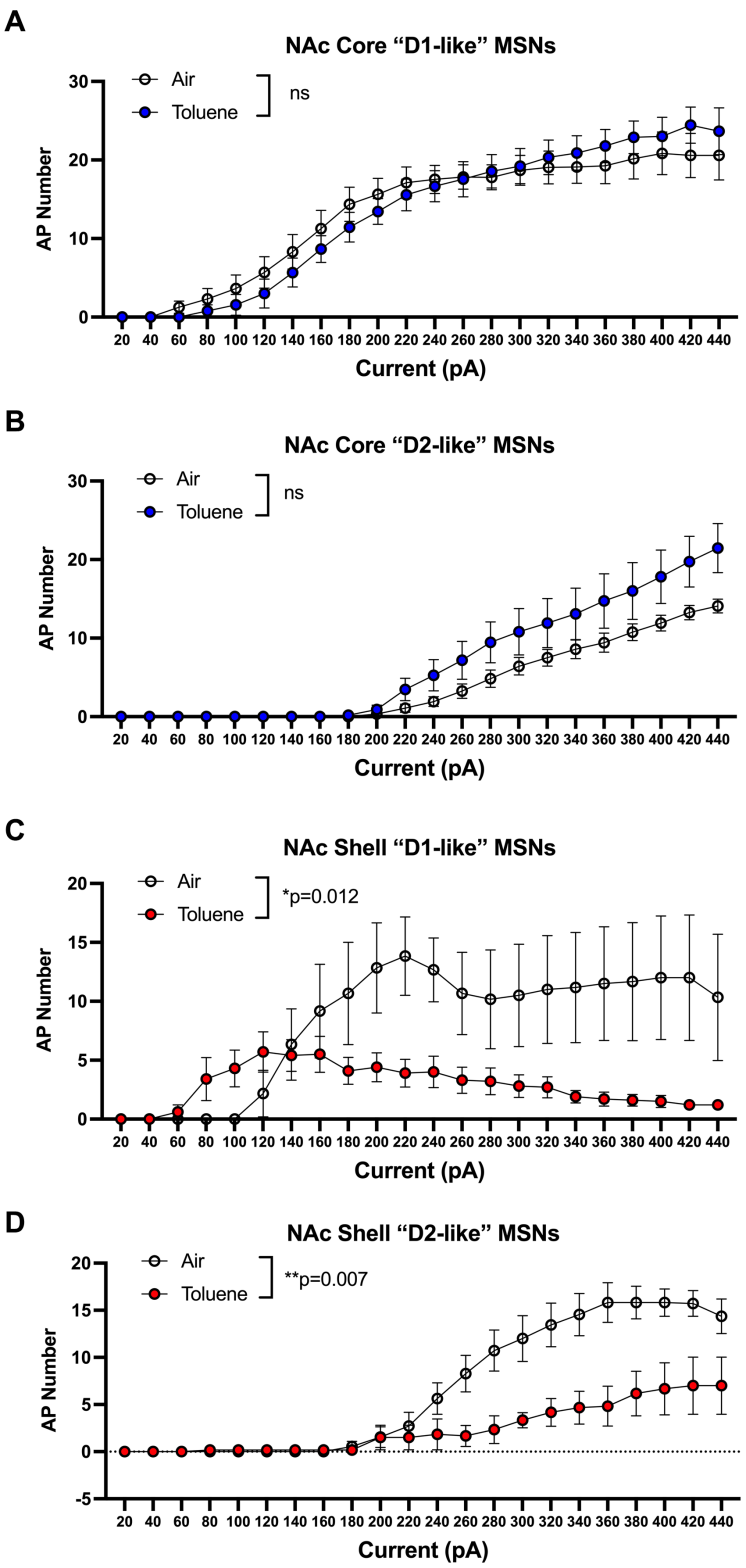
Effects of toluene vapor exposure on “D1-like” and “D2-like” MSNs in the NAc core and shell of adolescent Sprague–Dawley rats. MSNs were classified as “D1-like” or “D2-like” based on their rheobase (see Results section for details). Toluene exposure had no significant effect on spiking of NAc core “D1-like” **(A)** or “D2-like” **(B)** MSNs. **(C)** Compared to air controls, exposure to toluene vapor enhanced spiking of NAc shell “D1-like” MSNs at low current steps and reduced spiking at higher current steps (RM two-way Anova *F*(_1,14_) = 8.22, *p* = 0.012). **(D)** Toluene vapor reduced spiking of NAc shell “D2-like” MSNs (RM two-way Anova *F*(_1,15_) = 9.76, *p* = 0.007). Data are presented as mean ± sem.

In the first study, adolescent (PND 41–44; Figure 1A timeline) male Sprague–Dawley rats were exposed to air or toluene vapor using a protocol that mimics human voluntary solvent inhalation. This consisted of two 10 min exposures to 10,500 ppm toluene vapor separated by a 10 min air exposure and a final 20 min air washout. Twenty-four hours later, animals were sacrificed and current-evoked action potentials (AP) were generated in medium spiny neurons located in the core and shell of the nucleus accumbens. As shown in Figure 1B, AP firing of NAc core neurons was similar between air and toluene exposed animals (RM two-way Anova *F*(_1,53_) = 0.052, *p* = 0.82) with AP number increasing as a function of the injected current. In contrast, there was a main effect of toluene exposure on NAc shell neuron firing (RM two-way Anova *F*(_1,27_) = 11.65, *p* = 0.002) with a leftward shift in the current-firing relationship of NAc shell neurons from toluene treated animals with reduced firing at higher current injections as neurons went into depolarization block (Figure 1C). When summed over all current injections, the total number of spikes generated by NAc shell MSNs from control animals was significantly greater than that from the toluene treated animals (mean ± sem; Air 135.3 ± 18.7; Toluene 61.8 ± 10.58; *t* = 3.43, df = 26, *p* = 0.002, unpaired *t*-test). NAc medium spiny neurons are classified into two major subtypes based on their expression of D1 or D2 dopamine receptors (Matamales et al., 2009). In a previous study, we showed that the subtype of an MSN in the adolescent rat NAc could be putatively assigned based on electrophysiology parameters with D1 MSNs requiring less current to begin firing (measured as the rheobase) than D2 MSNs (Beckley et al., 2016). Using this method, we classified MSNs as “D1-like” when firing was induced at currents less than 180 pA (NAc core) or 200 pA (NAc shell) while those having a rheobase higher than these values were classified as “D2-like.” We then replotted the air and toluene current-spiking curves for “D1-like” and “D2-like” MSNs for NAc core and NAc shell. As shown in Figure 2A, toluene had no effect on AP firing in NAc core “D1-like” MSNs (RM two-way Anova *F*(_1,26_) = 0.002, *p* = 0.96) and produced a small but statistically insignificant increase in firing of NAc core “D2-like” neurons (RM two-way Anova *F*(_1,21_) = 3.11, *p* = 0.09; Figure 2B). In the NAc shell, this classification scheme showed that “D1-like” MSNs from toluene exposed rats had a reduced rheobase but less overall spiking than controls (RM two-way Anova *F*(_1,14_) = 8.22, *p* = 0.012; Figure 2C) while “D2-like” MSNs showed a significant reduction in spiking with no change in rheobase (RM two-way Anova *F*(_1,15_) = 9.76, *p* = 0.007; Figure 2D).

**Figure 3:**
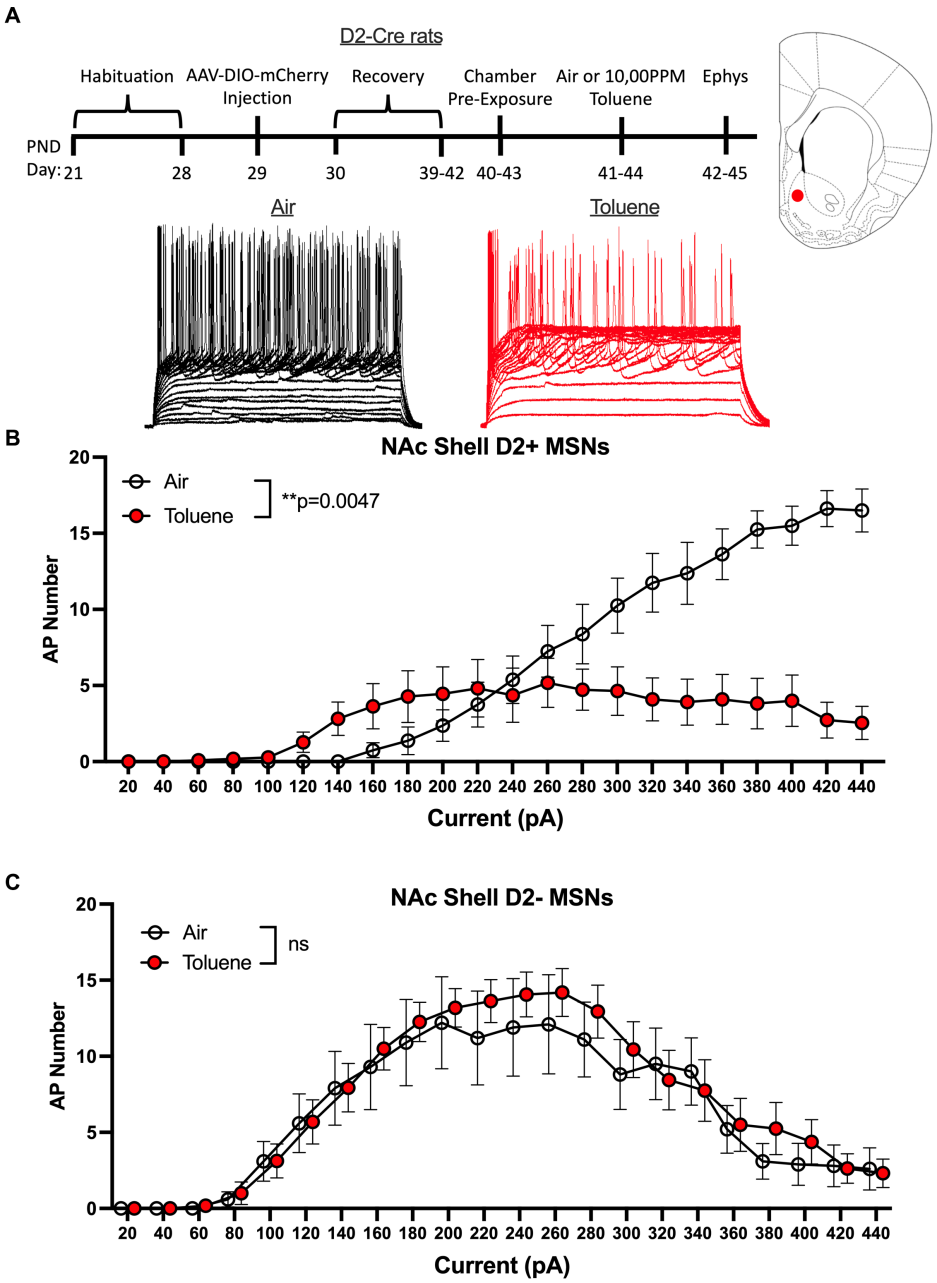
Exposure to toluene vapor alters the excitability of NAc shell D2+ MSNs in adolescent D2-Cre Long-Evans rats. **(A)** Schematic shows timeline of study and age of rats at viral surgery, toluene exposure and electrophysiology recordings. Inset shows approximate location of recordings for NAc shell MSNs (red dot). Traces show examples of current evoked spiking of NAc shell D2+ MSNs from air or toluene exposed rats. **(B)** Compared to air controls, toluene exposure increased firing of D2+ MSNs at low current steps and reduced spiking at higher current steps (RM two-way Anova *F*(_1,17_) = 10.55, *p* = 0.0047). **(C)** Toluene vapor had no effect on current-evoked spiking of NAc shell D2- MSNs. Data are presented as mean ± sem.

A limitation to this classification scheme is the possibility that toluene exposure may itself produce a change in the rheobase that would result in mislabeling of MSNs as D1 or D2. To address this issue, we conducted additional studies using rats that express Cre-recombinase under control of the D2 dopamine receptor promoter. To visually identify MSN subtypes in these animals during recordings, we infused an AAV virus containing a floxed mCherry reporter and targeted the NAc shell based on findings obtained with the Sprague–Dawley rats. After recovery, rats were exposed to air or toluene vapor at PND 40–43 and recordings were performed as described above (Figure 3A timeline). Recordings targeted mCherry positive (termed D2+ MSNs) and negative (termed D2- MSNs) neurons located adjacent to one another and were interleaved during daily recording sessions to ensure that experimental conditions were similar for both cell types. Current evoked firing of MSNs in air treated animals showed the expected difference in rheobase with D2+ MSNs requiring approximately twice as much current to begin firing than D2- MSNs. In NAc shell D2+ neurons, there was a main effect of toluene vapor exposure on current-induced firing (RM two-way Anova *F*(_1,17_) = 10.55, *p* = 0.0047) with a leftward shift in current-evoked firing that was accompanied by reduced spiking at higher current injections as compared to the air controls (Figure 3B). Summing over all current injections, the total number of spikes generated by NAc shell D2+ MSNs from control animals was significantly greater than that from the toluene treated animals (mean ± sem; Air 136.4 ± 18.1; Toluene 71.1 ± 16.4; *t* = 2.63, df = 15, *p* = 0.019, unpaired *t*-test). Spike firing of NAc shell D2- MSNs from air treated rats displayed a prominent biphasic current-spiking relationship with enhanced firing during initial current steps followed by reduced firing at higher current injections as neurons went into depolarization block (Figure 3C). A similar pattern was observed for D2- MSNs from toluene exposed animals and this was not different from the air controls.

**Figure 4:**
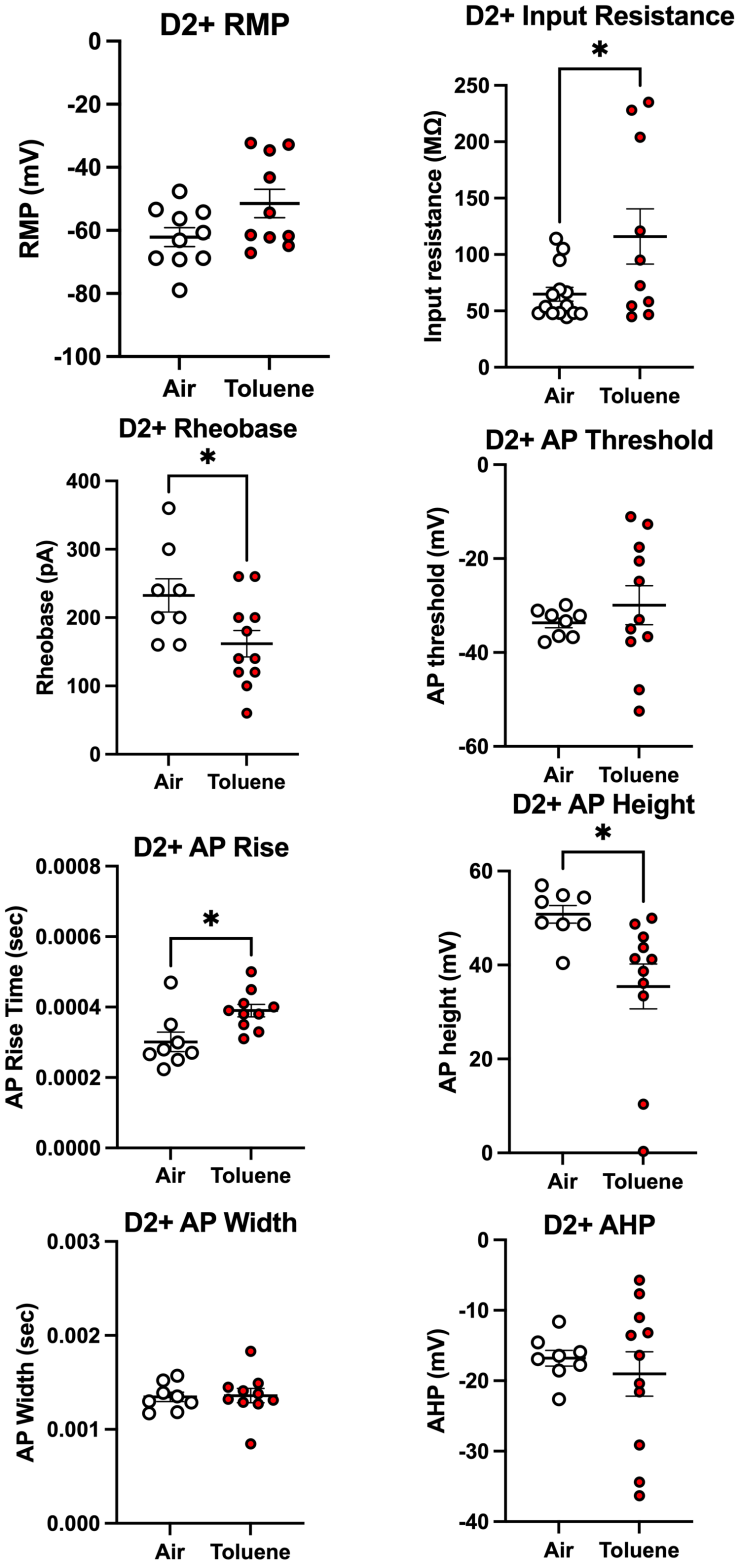
Toluene vapor alters the electrophysiological parameters of NAc shell D2+ MSNs. Exposure of D2-Cre rats to toluene vapor produced significant effects on input resistance (unpaired *t*-test; *t* = 2.35, df = 22, *p* = 0.028), rheobase (unpaired *t*-test; *t* = 2.30, df = 17, *p* = 0.035), AP rise time (unpaired *t*-test; *t* = 2.83, df = 16, *p* = 0.012) and AP height (unpaired *t*-test; *t* = 2.62, df = 17, *p* = 0.018) but no effect on resting membrane potential, AP threshold, AP width or after-hyperpolarization (AHP) of D2+ MSNs. Data are presented as mean ± sem. Symbol: (*) value significantly different from air control group *p* < 0.05.

**Figure 5:**
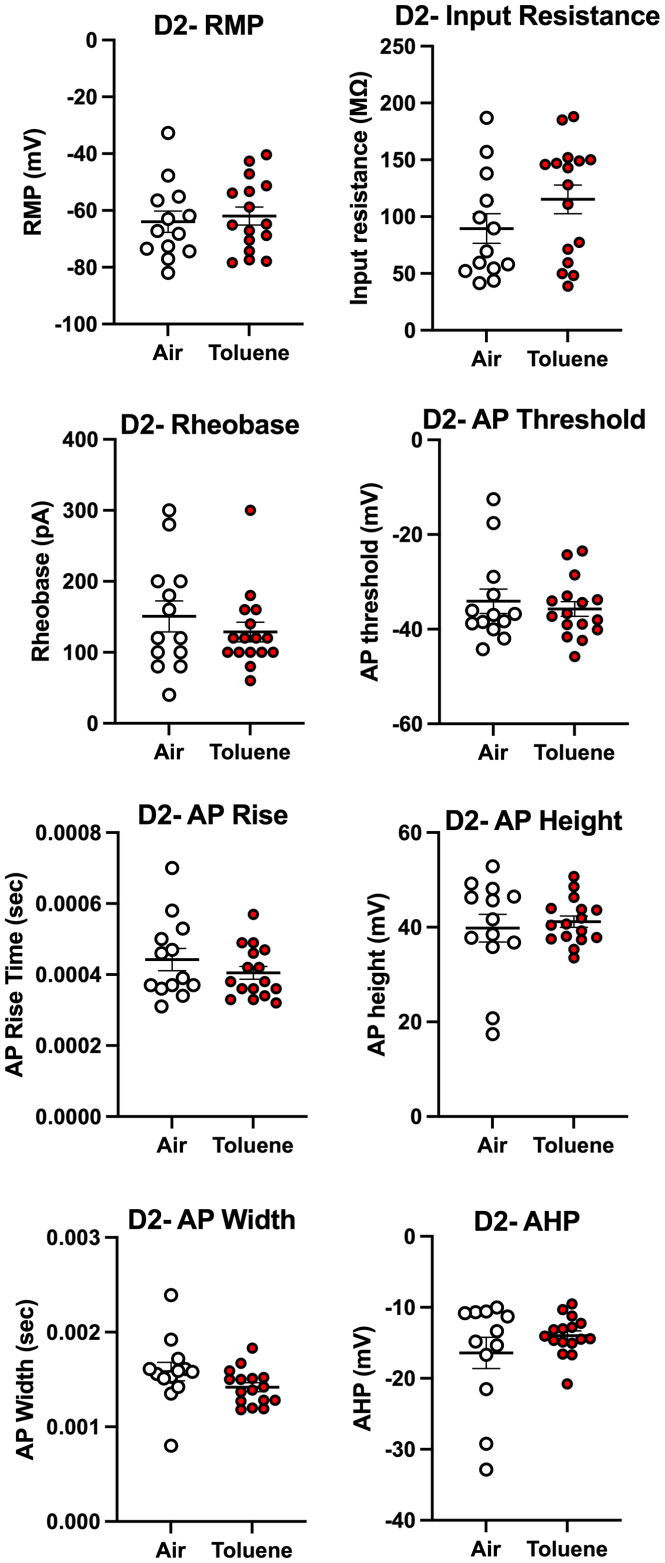
Exposure to toluene vapor had no effect on electrophysiological parameters of NAc shell D2- MSNs. Data are presented as mean ± sem.

To assess whether toluene exposure induced any changes in basic neuronal properties and action potential characteristics, we measured a variety of electrophysiological parameters using recordings with current steps that generated 4–5 spikes. As shown in Figure 4, toluene exposure increased the input resistance (unpaired *t*-test; *t* = 2.35, df = 22, *p* = 0.028) and decreased the rheobase (unpaired *t*-test; *t* = 2.30, df = 17, *p* = 0.035) of NAc shell D2+ MSNs as compared to air controls with no change in resting membrane potential or AP threshold. Toluene exposure also increased the AP rise time (unpaired *t*-test; *t* = 2.83, df = 16, *p* = 0.012) and reduced total AP height (unpaired *t*-test; *t* = 2.62, df = 17, *p* = 0.018) in NAc shell D2+ MSNs but had no effect on AP width after-hyperpolarization (AHP). Figure 5 shows the neuronal properties and AP characteristics of NAc shell D2- MSNs from air or toluene treated rats. Consistent with the findings from the current-evoked firing recordings, there were no significant differences in electrophysiological parameters of NAc shell D2- MSNs between air and toluene treated animals.

## Discussion

The major finding of this study is that a brief exposure to a binge-like concentration of toluene vapor that mimics voluntary inhalant use in humans alters the excitability of NAc medium spiny neurons in a sub-region and cell-type dependent manner. In Sprague–Dawley rats, toluene vapor had no effect on the excitability of MSNs in the NAc core but produced a leftward and downward shift in the intrinsic excitability of MSNs in the NAc shell. Using D2-Cre reporter rats, this effect was found to be restricted to NAc shell D2+ MSNs and was accompanied by changes in electrophysiological parameters recorded from these neurons. These findings are consistent with and add to findings from previous studies reporting selective effects of toluene on neurons within the addiction neurocircuitry that vary based on brain region, neuronal cell-type and projection target (Beckley et al., 2016; Wayman and Woodward, 2018b).

The mechanism underlying the selective effect of toluene on D2+ MSN intrinsic excitability observed in this study is currently unknown. In a previous study, a brief bath application of 0.3 or 3 mM toluene had minimal effects on current-evoked firing of NAc MSNs although those recordings primarily targeted neurons located in the NAc core (Beckley et al., 2016). Toluene, at 3 mM, did slightly reduce the fast after-hyperpolarization of these neurons and results using pharmacological antagonists showed that these currents are likely generated by members of the BK family of potassium channels (Beckley et al., 2016). In contrast to the lack of effect of toluene on current-evoked spike firing reported in that study, toluene produced a long-term depression (LTD) of AMPA-mediated synaptic currents in approximately 50% of the MSNs. These neurons were identified as D2 MSNs via immunocytochemistry and the toluene-induced LTD was prevented when recordings were done in the presence of the cannabinoid type 1 receptor antagonist AM-281 suggesting a role of endocannabinoids (eCB). A similar EC-mediated LTD of AMPA synaptic signaling was observed in glutamatergic pyramidal neurons in the mPFC and basolateral amygdala following bath application of toluene (Beckley and Woodward, 2011; Woodward, 2023). D2 MSNs in the NAc have been shown to undergo eCB-mediated LTD (Grueter et al., 2010) as well as depolarization-induced suppression of excitation (DSE) that also involves endocannabinoid signaling (Kreitzer and Regehr, 2001; Beckley et al., 2016). Whether *in vivo* exposure to toluene vapor induces LTD or DSE in NAc D2 MSNs is not known but these events could trigger compensatory changes that underlie the increase in intrinsic excitability of D2 NAc shell MSNs observed during injections of low to moderate amounts of current. Consistent with this change in excitability was the increase in membrane resistance observed in D2+ NAc shell MSNs from toluene treated rats and the corresponding reduction in the rheobase. Despite the leftward shift in the current-spiking relationship in NAc shell D2+ MSNs in toluene treated animals, these neurons went into depolarization block and were unable to increase their firing as larger currents were injected. This may have resulted from toluene-induced alterations in expression or function of the voltage-gated sodium and potassium channels that generate the action potential (Bianchi et al., 2012; Knowlton et al., 2021). Analysis of the action potential parameters revealed a significant decrease in action potential height and a slower rise time in NAc shell D2+ MSNs from toluene treated rats with no changes in these parameters in NAc shell D2- MSNs. Although NAc shell D2+ MSNs from toluene treated rats began firing at lower current steps than the air controls, their inability to increase spiking at higher current injections could have important consequences in control of motivated behavior as these neurons receive dense inputs from various structures including prelimbic and infralimbic regions of the mPFC that have been shown to regulate drug seeking and reinstatement of drug seeking following extinction (Peters et al., 2008; Bossert et al., 2012; Allichon et al., 2021).

Previous studies using *in vivo* toluene exposure protocols have reported changes in current-evoked firing of pyramidal neurons within the mPFC including those that project to different areas of the NAc. For example, repeated exposures of adolescent Wistar rats to toluene vapor (1,000–8,000 ppm, 30 min twice per day for 10 days) that induces CPP enhanced the excitability of deep-layer pyramidal neurons in the prelimbic area of the mPFC (Cruz et al., 2020). In another study, retrobeads were used to identify inputs to the NAc core and shell from the mPFC in adolescent Sprague–Dawley rats. One day following exposure to 10,500 ppm toluene vapor, current-evoked firing of deep layer prelimbic mPFC neurons that project to the NAc core was reduced while firing was enhanced in NAc core projecting deep layer infralimbic mPFC neurons (Wayman and Woodward, 2018b). In contrast, there was no effect of toluene exposure on NAc shell projecting prelimbic mPFC neurons but firing was reduced in NAc shell projecting infralimbic mPFC neurons. These findings suggest that in addition to changes in local mechanisms that control the intrinsic excitability of NAc MSNs, toluene-induced alterations in inputs to the NAc from the mPFC and other areas could contribute to the changes in the excitability of NAc MSNs noted in the present study. The ultimate behavioral consequences of these changes are not fully known but may contribute to the development of reward-related memories following repeated exposures to drugs of abuse including inhalants. For example, adolescent rats given repeated exposures to air or toluene vapor in different compartments displayed a conditioned place preference (CPP) that persisted for at least 7 days following the last pairing (Wayman and Woodward, 2018a). The toluene-induced CPP was associated with opposing changes in the firing of infralimbic mPFC neurons projecting to the NAc core (increased) or shell (decreased) and was blocked by chemogenetic excitation of infralimbic NAc shell projecting neurons (Wayman and Woodward, 2018a). Together with the findings of the present study, these results suggest that reduced activity of the infralimbic mPFC-NAc shell D2 pathway may be especially important in driving the rewarding aspects of toluene vapor. It is important to note that most preclinical studies of toluene action including the present one have used male rats (reviewed by Crossin and Arunogiri, 2020). As human adolescent females also engage in inhalant misuse (SAMHSA, 2021), future preclinical studies should include both males and females to investigate possible sex-dependent differences in the effects of toluene vapor on neural signaling in addiction related brain areas.

## Data availability statement

The raw data supporting the conclusions of this article will be made available by the authors, without undue reservation.

## Ethics statement

The animal studies were approved by Institutional Care & Use Committee (IACUC); Medical University of South Carolina. The studies were conducted in accordance with the local legislation and institutional requirements. Written informed consent was not obtained from the owners for the participation of their animals in this study because animals were purchased from commercial vendors.

## Author contributions

JW was responsible for the conception, design of the study, and wrote the first draft of the manuscript with contributions from MO and AK. MO, AK, and DG conducted the electrophysiology studies. MO and JW analyzed the electrophysiology data and JW performed the statistical analysis of the data. All authors contributed to the article and approved the submitted version.

## Funding

This work was supported by NIH grants R01DA013951 (JW), T32AA007474 (JW) and F32AA026774 (DG).

## Conflict of interest

The authors declare that the research was conducted in the absence of any commercial or financial relationships that could be construed as a potential conflict of interest.

## Publisher’s note

All claims expressed in this article are solely those of the authors and do not necessarily represent those of their affiliated organizations, or those of the publisher, the editors and the reviewers. Any product that may be evaluated in this article, or claim that may be made by its manufacturer, is not guaranteed or endorsed by the publisher.
